# Structured Exercise Modulates Gut Microbiota Composition and Protects Against Diet-Induced Dysbiosis in a Rat Model

**DOI:** 10.3390/nu18050847

**Published:** 2026-03-05

**Authors:** Fatiha M. Benslimane, Maha Alser, Abdelrahman M Elgamal, Layla I. Mohammed, Zain Zaki Zakaria, Sara Sokary, Muhammad Umar Sohail, Ayat S Hammad, Saddam Akber Abbasi, Maha Al-Asmakh

**Affiliations:** 1 Biomedical Research Center, QU Health, Qatar University, Doha P.O. Box 2713, Qatar; fatiha@qu.edu.qa (F.M.B.);; 2 Department of Biomedical Sciences, College of Health Science, QU Health, Qatar University, Doha P.O. Box 2713, Qatar; 3 Medical and Health Sciences Office, QU Health, Qatar University, Doha P.O. Box 2713, Qatar; 4 PremieHealth Pty Ltd., Level 11, CSL Global Headquarters, 655 Elizabeth Street, Melbourne, VIC 3000, Australia; 5 Statistics Program, Department of Mathematics and Statistics, College of Arts and Sciences, Qatar University, Doha P.O. Box 2713, Qatar

**Keywords:** high-fat diet, exercise, microbiota, Oxford Nanopore Technologies, *Akkermansia*

## Abstract

**Background/Objectives:** Dietary composition and physical activity are major determinants of gut microbiome structure, and dysbiosis is strongly associated with metabolic disorders. While both diet and exercise independently influence the gut microbiome, their interactive effects—particularly across different exercise modalities—remain incompletely understood. This study investigated the combined effects of diet type (normal chow [NC] vs. high-fat diet [HFD]) and exercise modality (control [C], voluntary [V], and forced [F]) on gut microbiota composition in rats. **Methods:** Sixty-three Wistar rats were randomized into six groups according to diet and exercise status. Fecal samples were collected and analyzed using full-length 16S rRNA gene sequencing (Oxford Nanopore Technologies). Alpha and beta diversity metrics were calculated, and taxonomic composition was assessed at phylum and genus levels. **Results:** HFD groups exhibited significantly higher alpha diversity than NC groups (Shannon index: 3.47–3.63 vs. 2.76–2.94, *p* < 0.001), with forced exercise associated with a greater diversity than voluntary exercise. Beta-diversity analysis confirmed diet as the dominant factor influencing microbial structure (PERMANOVA *p* = 0.001), with exercise providing an additional modulatory effect. Firmicutes, Bacteroidota, Deferribacterota, and Proteobacteria predominated, with Firmicutes decreasing under HFD. Forced exercise significantly enriched beneficial genera, including *Akkermansia* (detected exclusively in exercised HFD groups; *p* = 0.03), *Blautia*, *Coprococcus*, and *Roseburia*. *Akkermansia* abundance correlated positively with exercise distance (*p* < 0.001) and negatively with body weight (*p* < 0.01). **Conclusions:** Structured exercise, particularly forced treadmill training, attenuates HFD-associated dysbiosis and promotes the beneficial gut bacteria that is associated with metabolic health. These findings highlight exercise modality as a critical factor in dietary strategies targeting gut microbiome modulations.

## 1. Introduction

The mammalian gastrointestinal tract harbors approximately 10–100 trillion microorganisms [1,2], collectively referred to as gut microbiota, which play pivotal roles in host physiology, including energy homeostasis, metabolic regulation, and endocrine function [3,4]. These microorganisms contribute to host defense by competing with pathogens for colonization niches and producing metabolites, such as short-chain fatty acids (SCFAs), which exhibit anti-inflammatory properties and enhance intestinal barrier integrity [4]. Dysbiosis of gut microbiota has been strongly associated with obesity, type 2 diabetes (T2D), and non-alcoholic fatty liver disease (NAFLD) [5]. Although factors such as dietary habits, antibiotic exposure, age, and sex are well-established modulators of gut microbiota composition [6], the role of physical activity—particularly exercise modality—within the context of dietary interventions remains poorly understood.

Obesity, defined by a body mass index (BMI) ≥ 30 kg/m^2^, substantially elevates the risk of T2D, cardiovascular disease, hypertension, and certain cancers [7]. The etiology of obesity and obesity-related metabolic disorders (ORMDs) is multifactorial, involving complex interactions between genetic predisposition and environmental factors [8,9]. Sedentary lifestyles, combined with high-fat, energy-dense diets, have contributed to the rising ORMDs, particularly in developed countries and the Gulf region [10]. High-fat diets are known to alter gut microbiota composition, promoting bacteria taxa positively associated with obesity and metabolic dysfunction [6,11,12,13]. Conversely, physical activity may mitigate these obesogenic effects and reshape gut microbial communities [14,15].

Emerging evidence suggests that exercise influences gut microbiota composition [16,17] through several putative mechanisms, including alteration in intestinal transit time, modulation of bile acid metabolism, enhanced SCFA production, immune regulation, and improved gut barrier function [18,19,20,21]. Exercise parameters, particularly those described by the FITT principle (frequency, intensity, time, and type), are thought to differentially modulate these mechanisms, with variations in exercise type and intensity producing distinct microbial responses [22]. However, most studies examine diet or exercise in isolation [18,19], and critical gaps remain in the understanding of how different exercise modalities interact with dietary patterns to shape the gut microbiome.

Voluntary and forced exercise represent two fundamentally distinct paradigms that differ not only in intensity and regularity but also in their neuroendocrine profiles. Forced treadmill exercise typically elicits higher corticosterone responses and provides standardized intensity, duration, and frequency, whereas voluntary wheel running varies considerably between individual animals in total distance, bout patterns, and associated physiological stress [23,24]. These distinctions may differentially influence gut physiology and microbial ecology, warranting their direct comparison. Despite this, the distinct effects of these exercise modalities on diet-induced microbial alterations are not well defined.

The Wistar rat model is well established for studying diet-induced metabolic dysregulation and offers the advantage of controlled dietary intake, standardized exercise protocols, and reduced genetic variability, making it particularly suitable for investigating diet–exercise–microbiota interactions [15,25]. Therefore, this study aimed to characterize the combined and interactive effects of diet type (normal chow [NC] versus high-fat diet [HFD]) and exercise modality (sedentary, voluntary wheel running, or forced treadmill exercise) on gut microbiota diversity, taxonomic composition, and the abundance of key bacterial taxa in rats.

## 2. Materials and Methods

### 2.1. Study Design and Sample Collection

All animals were housed and maintained in Qatar University’s Laboratory Animal Research Center (LARC). The study involved 63 Wistar rats, aged 10 weeks at study initiation, randomized into six experimental groups based on diet intake (NC and HFD) and exercise status (control [C], voluntary [V], and forced [F] exercise), as shown in Table 1. The study included both male and female Wistar rats. Preliminary analyses showed no significant sex-dependent differences in gut microbiota composition; therefore, data from both sexes were pooled for all subsequent analyses.

Rats were allocated to experimental groups using simple randomization. Due to the nature of the exercise interventions (treadmill vs. running and wheel vs. sedentary housing), blinding investigators during the intervention phase was not feasible. However, wet lab, bioinformatic processing and statistical analyses were performed without prior knowledge of group assignments.

Animals were housed in individually ventilated cages (IVC) under standard conditions: room temperature of 21–26 °C, relative humidity of 40–65%, and 12/12 h light/dark cycle. Animals had free access to drinking water (ad libitum) throughout the study. Animals were fed either an NC diet or an HFD. The NC diet was SAFE diet 150 (SAFE, Augy, France; 3.4 kcal/g), consisting of 21% protein, 12.6% lipids, and 66.4% carbohydrates. The HFD corresponded to diet 260HF (U8978, version 19; SAFE, Augy, France), a high-fat, high-sucrose diet widely used to induce metabolic dysfunction in rodent models. This diet provides approximately 36% of total energy from fat, 20% from protein, and 37% from carbohydrates, including 14.5% starch and 17.9% sucrose.

Exercise protocols: Following one week of acclimatization, the F groups underwent treadmill running on a motor-driven rodent treadmill (EXER-3/6, Columbus Instruments, Columbus, OH, USA) at 18–20 m/min for 30 min per session, 5 days per week for 8 weeks [26,27]. The V groups were housed in cages with free 24 h access to telemetered running wheels for the same duration. Control groups remained sedentary in standard cages. To control the variable of handling stress, all groups were handled equally on intervention days.

Fecal samples were collected from the cecum post-sacrifice for microbiome analysis. The animal study protocol was approved by the Institutional Animal Care and Use Committee (IACUC) of Qatar University (protocol code QU-IACUC 007/2020). The study adhered to all relevant national and international regulations and guidelines for research involving animal subjects and is reported to comply with local and international guidelines.

Body weight was recorded at the study initiation and at the end of the 8-week intervention period. As the primary objective of this study was to characterize gut microbiota composition, additional metabolic and physiological parameters such as food intake, glucose levels, and adiposity indices were not assessed.

### 2.2. DNA Extraction

Total DNA was extracted from fecal samples using the DNeasy Kits (Qiagen^®^, Hilden, Germany) according to the manufacturer’s recommendations [28]. DNA concentration and purity were assessed using a Nanodrop spectrophotometer (Thermo Fisher Scientific, Waltham, MA, USA) and the Qubit dsDNA High Sensitivity Assay kit (Thermo Fisher Scientific, USA, Cat. No. Q32854).

### 2.3. Library Preparation and Sequencing

Ten nanograms of high-quality total DNA from each sample were used for library preparation using the Oxford Nanopore Technologies (ONT) 16S Barcoding Kit (SQK-16S024). Full-length 16S rRNA gene amplification was performed using LongAmp^®^ Hot Start Taq 2X Master Mix (New England Biolabs, Ipswich, MA, USA), with the universal primer set to 27F/1492R containing 5′ tags to facilitate the ulterior ligase-free attachment of the sequencing adapters.

PCR amplicons were purified using CleanMag^®^ Magnetic Beads (Paragon Genomics, Fremont, CA, USA) at a 0.6× bead-to-sample ratio. Purified, barcoded libraries were quantified and pooled at equimolar concentrations (60 fmol per sample) in an elution buffer (pH 8.0). The rapid sequencing adapter (RAP) was added to the pooled libraries and incubated for 5 min at room temperature, followed by the addition of a sequencing buffer (SQB) and loading beads (LB). The libraries were then loaded onto a Flongle flow cell R9.4.1 (FLO-FLG001). The sequencing was launched under high-accuracy basecalling parameters with a minimum Q-Score threshold of >7. Basecalling was performed using MinKNOW software (v3.6.5).

### 2.4. Bioinformatics and Statistical Analysis

Raw sequencing data (fastq_pass files) were analyzed using EPI2ME software (v5.1.3) using the 16S workflow (wf-16S, v0.0.3). Sequence alignment was conducted using minimap2 (v2.26-r1175) against the NCBI 16S rRNA database. Taxonomic classification was performed by aligning full-length 16S rRNA gene sequences (~1500 bp) to the NCBI 16S rRNA database using minimap2, under strict quality filtering criteria including high-accuracy basecalling, a minimum Q-score of >9, and a retention of reads between 1300 and 1700 bp only. Species-level classification was assigned at a ≥97% sequence identity and ≥95% query coverage threshold, which are widely accepted standards for species-level taxonomic assignment in 16S rRNA-based studies. Combined with full-length sequencing of the complete 16S rRNA gene, which provides substantially higher resolution than short-read approaches targeting individual hypervariable regions, this approach enables robust taxonomic classification at both genus and species levels. Taxonomic count tables (CSV files) were exported from EPI2ME, and the relative abundance was calculated. Taxa with a relative abundance of ≥5% were retained for downstream analysis.

Statistical analysis was performed using the R programming language (v4.3.1, 2023). Alpha diversity indices (Sobs and Shannon) and beta diversity (Bray–Curtis dissimilarity) were computed using the vegan package (v2.6.4) [29]. Statistical differences in microbial diversity among groups were assessed using the Kruskal–Wallis test and Dunn’s post hoc test. The aligned rank transformation ANOVA (ART ANOVA; v0.11.1) was applied to evaluate the significance among microbial communities across exercise and diet. This is a nonparametric approach that allows for multiple independent variables, interactions, and repeated measures [30,31]. A Tukey-adjusted contrast test was implemented using ART as a post hoc test [30]. Spearman’s correlation analysis was used to assess the association between genus-level microbial abundance and clinical parameters. Data visualization was performed using ggplot2 (v3.4.3). For visualization purposes only, taxa with a relative abundance of <1% were excluded to improve graphical clarity; however, all statistical analyses were conducted on the complete unfiltered dataset. Statistical significance annotations were added using the ggsignif package (v3.4.3). A *p*-value of ˂ 0.05 was considered statistically significant.

## 3. Results

This section summarizes the effects of diet type and exercise modality on body weight, physical activity, and gut microbiota composition in the experimental rat groups.

### 3.1. Characteristics of Study Animal Subjects

At study initiation, mean body weights were comparable between the dietary groups (NC: 94.1 ± 0.8 g and HFD: 91.3 ± 4.3 g). By the end of the intervention, animals in both groups exhibited approximately a twofold increase in body weight, reaching similar final values (NC: 190.6 ± 4.7 g and HFD: 189.3 ± 5.3 g). The mean daily running distance differed substantially between the exercise modalities. The rats in the voluntary exercise groups demonstrated markedly greater but highly variable spontaneous activity (V-NC: 24.55 ± 30.71 km/day and V-HFD: 6.40 ± 7.79 km/day) compared with the standardized forced treadmill groups (F-NC 0.18 ± 0.005 km/day and F-HFD: 0.18 ± 0.005 km/day). Notably, high-fat feeding was associated with reduced voluntary running distance relative to normal chow. Across exercise modalities, rats subjected to voluntary exercise (V-NC, V-HFD) demonstrated lower overall weight gain compared with those undergoing forced treadmill exercise (F-NC, F-HFD). The differences in the mean weight gain were approximately 23 g between the V-NC and F-NC groups and ~13–14 g between the V-HFD and F-HFD groups. These findings indicate a trend toward attenuated weight gain with voluntary exercise relative to forced exercise, irrespective of dietary condition.

### 3.2. The Effect of Diet and Exercise on Bacterial Diversity and Community of Gut Microbiota

To evaluate how diet and exercise influence gut microbial diversity and community structure, we analyzed alpha and beta diversity metrics across the six experimental groups using 16S rRNA sequencing data.

#### 3.2.1. Alpha Diversity

**Figure 1:**
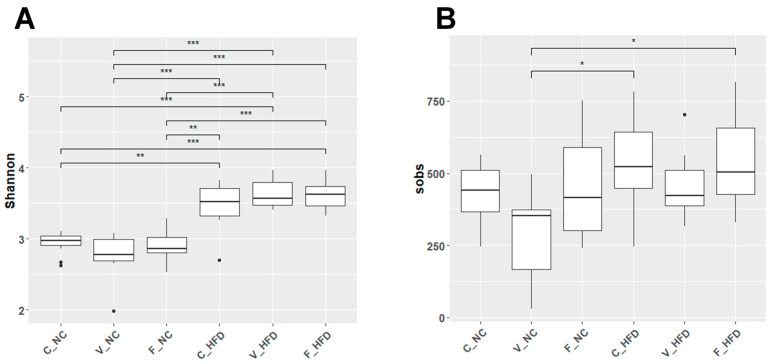
Microbial alpha diversity across dietary and exercise groups. Boxplots of bacterial species diversity richness and evenness using (**A**) Shannon diversity index and (**B**) Sobs value. Statistical significance was calculated using an aligned rank transformation ANOVA (ART ANOVA) with a post hoc Tukey-adjusted contrast test. Significance levels are denoted as * *p* < 0.05, ** *p* < 0.01, and *** *p* < 0.001. The plots represent the six experimental groups, C-NC (control–normal chow), V-NC (voluntary exercise–normal chow), F-NC (forced exercise–normal chow), C-HFD (control–high-fat diet), V-HFD (voluntary exercise–high-fat diet), and F-HFD (forced exercise–high-fat diet).

Alpha diversity was assessed across the six rat groups using the Shannon and Sobs indices (Figure 1). The Shannon index reflects both species’ richness and evenness. Diet type exerted the strongest effect on gut microbial diversity. A statistically significant increase in Shannon diversity was observed in all HFD groups compared with their corresponding NC groups. Table 2 summarizes the mean ± SD values for each group. Shannon index values ranged from 2.76 to 2.94 in the NC groups and 3.47–3.63 in the HFD groups. The lowest Shannon index was observed in the V-NC group (2.76 ± 0.33), whereas the highest was detected in the V-HFD group (3.63 ± 0.19 and *p* = 8.78 × 10^−5^). Similarly, species richness was further assessed using the Sobs index. The lowest Sobs value was observed in the V-NC group (299.7 ± 149.8), while the highest was found in the F-HFD group (547.7 ± 170.2). All HFD groups consistently exhibited greater species richness than their NC counterparts. Notably, the observed increase in alpha diversity under HFD conditions does not necessarily indicate improved microbial health; this finding is further interpreted in the context of dysbiosis in the Discussion. Overall, these findings indicate that an HFD increases gut microbial diversity and richness, while exercise further modulates these effects, with forced exercise exerting a stronger influence than voluntary exercise.

#### 3.2.2. Beta Diversity

**Figure 2:**
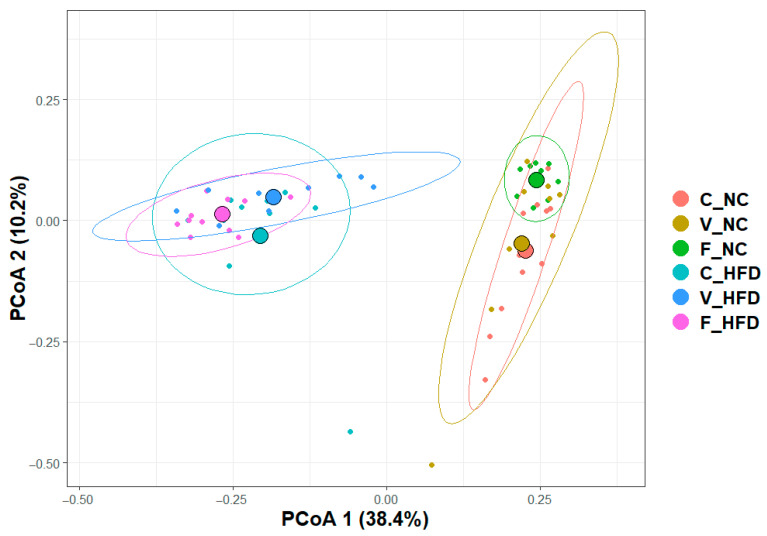
Microbial beta-diversity. Principal coordinates analysis (PCoA) of the Bray–Curtis Dissimilarity matrix comparing microbial community composition across the six experimental groups: C-NC (control–normal chow), V-NC (voluntary exercise–normal chow), F-NC (forced exercise–normal chow), C-HFD (control–high-fat diet), V-HFD (voluntary exercise–high-fat diet), and F-HFD (forced exercise–high-fat diet), which are color coded on the right. PERMANOVA was implemented to assess statistical significance (*p* = 0.000999001). The axes, PCoA 1 (38.4%) and PCoA 2 (10.2%), explain the variation in microbial community structure between the groups. Each point represents an individual rat, and the colored ellipses highlight the clustering patterns for each group. The groups are color coded as shown on the right of the plot.

Beta diversity was assessed using the Bray–Curtis dissimilarity index to evaluate differences in microbial community composition among the experimental groups. These differences are visualized using principal coordinates analysis (PCoA), as shown in Figure 2. PERMANOVA analysis demonstrated a highly significant effect (*p* = 0.001), indicating that both diet type and exercise significantly influence gut microbiota composition. A clear separation was observed between the two diet groups. The HFD groups clustered distinctly on the right side of the PCoA plot, whereas the NC groups clustered on the left. This separation highlights the dominant effect of diet on microbial community structure.

Sub-clustering within both diet groups further indicated the effect of exercise. Within the HFD groups, the centroids of each exercise subgroup were clearly separated, reflecting exercise-dependent modulation of the microbiome. In contrast, within the NC groups, the C and V groups showed substantial overlap, while the F group formed a distinct cluster. This suggests that forced exercise exerts a stronger influence on microbial composition under normal dietary conditions. Overall, these findings support the conclusion that diet is the primary driver of gut microbiota structure, while exercise modulates community composition in a context-dependent manner.

### 3.3. Taxonomy Analysis

To further characterize microbial community shifts induced by dietary intervention and exercise, taxonomic composition was examined at the phylum, genus, and species levels.

#### 3.3.1. Relative Mean Abundance of Gut Microbiota at the Phylum Level

**Figure 3:**
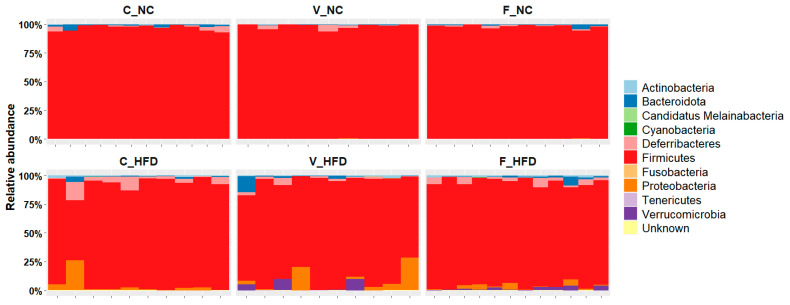
Taxonomy composition analysis at the phylum level. The bar charts represent the microbial relative mean abundance at the phylum level of the six experimental groups: C-NC (control–normal chow), V-NC (voluntary exercise–normal chow), F-NC (forced exercise–normal chow), C-HFD (control–high-fat diet), V-HFD (voluntary exercise–high-fat diet), and F-HFD (forced exercise–high-fat diet). Each bar represents a single rat within the group. The different phyla are color coded on the right of the plot.

Across the six experimental groups in Figure 3, 16S rRNA gene sequencing analysis identified ten known bacterial phyla and one unknown phylum. The rat gut microbiome was dominated by four major phyla with varying relative abundances (RA): Firmicutes, Bacteroidota, Deferribacteres, and Proteobacteria. Additional variability was observed in the distribution of Actinobacteria and Verrucomicriobia across groups (Table 3).

Actinobacteria were detected in four groups (C-NC, V-NC, F-NC, and C-HFD), whereas Verrucomicrobiota were exclusively present in the V-HFD and F-HFD groups. Firmicutes represented the dominant phylum in all groups, with mean relative abundances ranging from 86.5% to 98.0%. However, Firmicutes abundance was reduced in the HFD groups compared with the C-NC group.

In contrast, Bacteroidota were present at low relative abundances across all groups, with a modest increase observed in the V-HFD and F-HFD groups. The mean relative abundance of Deferribacterota was slightly reduced under HFD conditions; however, both voluntary and forced exercise appeared to partially restore its abundance. Actinobacteria and Proteobacteria showed increased relative abundances in the HFD groups, suggesting diet-associated shifts in microbial composition.

Notably, Verrucomicrobiota were detected only in the exercised HFD groups, with mean relative abundances of 2.6% (V-HFD) and 1.6% (F-HFD), indicating a potential exercise-dependent modulation of this phylum under high-fat dietary conditions.

#### 3.3.2. Relative Mean Abundance of Gut Microbiota at the Genus and Species Levels

**Figure 4:**
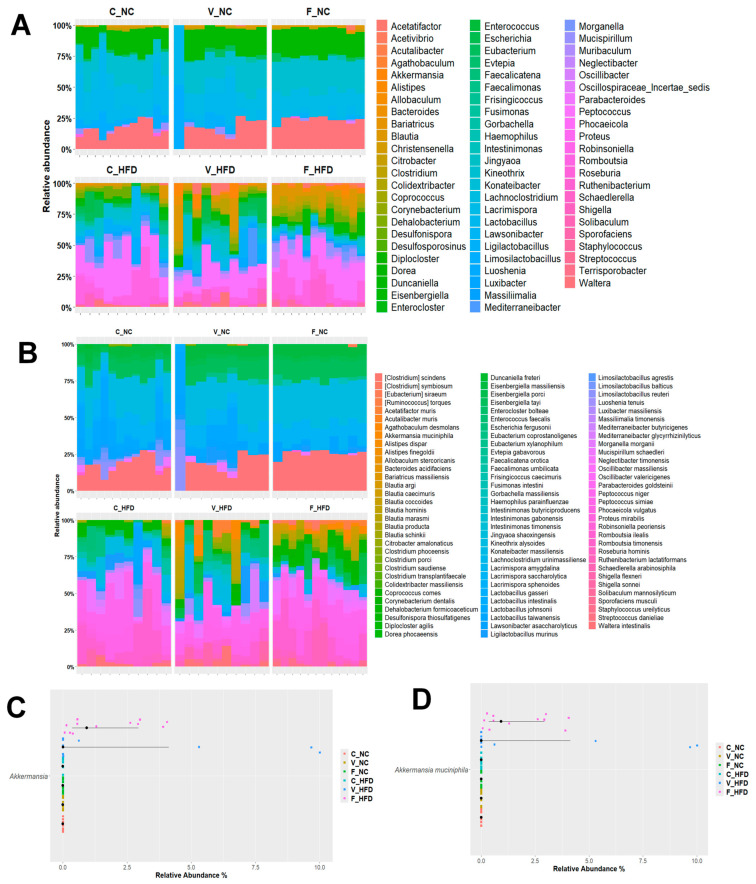
Taxonomic analysis of gut microbiota. Microbial relative mean abundance at the (**A**) genus and (**B**) species levels presented as bar charts. Each bar represents an individual rat within the experimental groups, with color segments corresponding to the relative abundance of bacterial taxa listed beside each chart. (**C**) Relative abundance of the genus Akkermansia and (**D**) the species *Akkermansia muciniphila* across experimental groups. Each dot represents an individual sample (rat). The experimental groups are: C-NC (control–normal chow), V-NC (voluntary exercise–normal chow), F-NC (forced exercise–normal chow), C-HFD (control–high-fat diet), V-HFD (voluntary exercise–high-fat diet), and F-HFD (forced exercise–high-fat diet), indicated by distinct colors.

We further analyzed the 16S rRNA gene sequencing data at the genus and species levels. Species-level classifications were assigned using full-length 16S rRNA gene sequences (~1500 bp) and strict criteria (≥97% sequence identity and ≥95% query coverage) against the NCBI 16S rRNA database, providing high-confidence taxonomic assignments. Based on this approach, a total of 508 genera were classified into 1020 species. For visualization purposes, Figure 4 presents taxa with a relative abundance of ≥1%, while all statistical analyses were performed using the complete dataset.

The five most abundant genera in each group are summarized in Supplementary Table S1. Under NC conditions, the dominant genera included *Lactobacillus*, *Kineothrix*, and *Eisenbergiella*. In contrast, HFD conditions were characterized by higher abundances of *Peptococcus*, *Romboutsia*, and *Blautia*. Exercise modulated these patterns, with both voluntary and forced exercise associated with an enrichment of genera linked to beneficial metabolic functions. Similarly, the five most abundant species per group are listed in Supplementary Table S2. *Kineothrix alysoides*, *Waltera intestinalis*, and *Lactobacillus johnsonii* predominated in the NC groups. Conversely, the HFD groups showed a higher abundance of *Peptococcus niger*, *Romboutsia ilealis*, and *Peptococcus simiae*. Exercise further influenced species-level composition, with *Ruthenibacterium lactatiformans* and *Desulfonispora thiosulfatigenes* detected exclusively in the F-HFD.

Members of the *Lactobacillus* genus were most abundant in NC-fed rats, whereas their relative abundance decreased in both voluntary and forced exercise groups under both dietary conditions. In contrast, the genus *Akkermansia* (phylum Verrucomicrobia) exhibited a notable response to diet and exercise. It was detected exclusively in the exercise HFD groups and showed a significant increase with both voluntary and forced exercise (*p* = 0.033), as shown in Figure 4A,C and summarized in Table 4. At the species level, *Akkermansia muciniphila* exhibited a significantly higher relative abundance in exercised HFD groups (*p* = 0.034; Figure 4D).

Diet and exercise also affected the abundance of several potentially pathogenic species. Several potentially pathogenic species were enriched in sedentary and voluntarily exercised HFD groups but were absent or markedly reduced in all other groups, including the F-HFD group (Table 5). These included *Shigella flexneri* (C-HFD: 0.89% and V-HFD: 1.45%), *Escherichia fergusonii* (C-HFD: 2.09% and V-HFD: 3.23%), *Shigella sonnei* (C-HFD: 0.38% and V-HFD: 0.58%), *Proteus mirabilis* (C-HFD only: 0.47%), and *Escherichia coli* (C-HFD: 0.08%; V-HFD: 0.15%; and *p*-adj < 0.01 for all comparisons). Notably, forced exercise under HFD conditions completely eliminated the detection of these species, suggesting a protective effect of structured exercise against diet-induced pathogenic enrichment.

### 3.4. Correlation Analysis

To explore potential associations between exercise parameters and microbiota composition, correlation analyses were performed between microbial taxa abundance and physiological variables within the experimental groups.

Correlation analyses were conducted in both voluntary and forced exercise HFD groups. Significant associations were detected only in the F-HFD group, likely due to the standardized exercise stimulus and lower inter-individual variability in running distance compared with voluntary exercise groups. Results from the voluntary exercise group were non-significant and are therefore not presented in detail. Our results revealed an increased relative abundance of the phylum Verrucomicrobiota in exercised groups (Figure 3), driven primarily by enrichment of the genus *Akkermansia* in exercised HFD groups compared with their non-exercised controls (Figure 4A). To further examine associations between exercise parameters and *Akkermansia* abundance, correlation analyses were performed.

**Figure 5:**
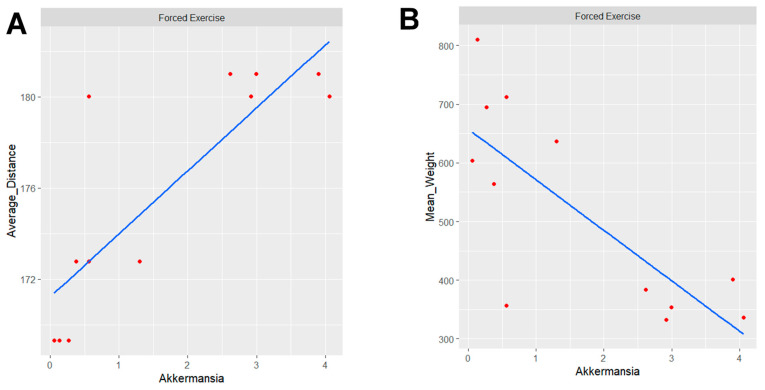
Spearman correlation analysis between the relative abundance of *Akkermansia* genera under forced exercise and (**A**) Average traveled distance and (**B**) Average rat weight.

Spearman correlation analysis was conducted within the F-HFD group. A significant positive correlation was detected between the average distance traveled and the relative abundance of *Akkermansia* in Figure 5A (*p* = 0.00029). Under forced exercise conditions, the traveled distance showed a positive correlation with the abundance of *Akkermansia* in the tested rat gut microbiota, with a *p*-value of 0.00029, suggesting that the longer the distance the rat travels, the higher the abundance of *Akkermansia*. We also examined the starting weight of the rats and its correlation with the distance traveled and *Akkermansia* abundance in Figure 5B. A significant negative correlation was observed between baseline body weight and *Akkermansia* abundance under forced exercise conditions in Figure 5B (*p* = 0.005801).

## 4. Discussion

The gut microbiota plays a fundamental role in host energy homeostasis, metabolic regulation, and immune function, with dysbiosis increasingly recognized as a central contributor to obesity and related metabolic disorders. While diet is widely acknowledged as the dominant environmental determinant of gut microbial composition, the independent and interactive effects of different exercise modalities remain incompletely understood. In this study, we demonstrate that diet and exercise interact synergistically rather than hierarchically to shape gut microbial diversity, composition, and functional potential. Although an HFD primarily dictated overall microbial structure, exercise modality critically modulated the abundance of specific bacterial taxa with established metabolic relevance. The biological significance of our key findings is strongly supported by the magnitude of observed differences. For example, Shannon diversity indices were consistently 0.5–0.9 units higher across all HFD groups compared to their NC counterparts, and Akkermansia was detected exclusively in exercised HFD groups with substantial relative abundances (V-HFD: 2.56% and F-HFD: 1.65%), demonstrating clear and biologically meaningful effects of both diet and exercise on gut microbial composition. These robust between-group differences, coupled with highly significant *p*-values, reinforce the strength of the observed diet–exercise–microbiota interactions.

Alpha diversity reflects ecosystem richness and evenness and is often interpreted as a marker of gut microbial health. In line with this, higher microbial alpha diversity has been associated with favorable metabolic outcomes in some contexts [32]. Our results demonstrated that both diet and exercise significantly influenced alpha diversity. Notably, HFD was associated with higher microbial diversity, richness, and evenness across all exercise conditions. While this finding appears to contradict studies linking plant-based, fiber-rich diets with elevated diversity [33,34,35], recent evidence suggests that HFD-induced diversity increases depend critically on dietary composition, particularly fiber content, which can vary substantially between commercially available high-fat formulations. The observed increase in alpha diversity under HFD likely reflects the proliferation of bacterial taxa specialized in lipid metabolism, as HFD created a selective environment favoring fat-utilizing bacteria while diminishing fiber-degrading populations. Consistent with this interpretation, we observed marked reductions in *Lactobacillus*, a genus typically enriched by dietary fiber [36], wit HFD reducing *Lactobacillus* abundance by up to 75% in some groups, mirroring findings by Lam et al. (2012) [37]. However, critically increased diversity under HFD does not equate to improved gut health. Our data revealed concurrent increases in Proteobacteria and potentially pathogenic species (*Shigella flexneri*, *Escherichia fergusonii*, and *Proteus mirabilis*), indicating that the elevated diversity observed here reflects dysbiosis rather than a healthy, balanced microbiome. This distinction between taxonomic diversity and functional microbial health is important, as compositional shifts favoring pathogenic or opportunistic taxa may drive metabolic dysfunction, systemic inflammation, and altered gut–brain axis signaling, despite an apparent increase in overall diversity [38].

The effects of exercise on overall alpha diversity were more modest than those of diet, which is consistent with systematic reviews ranking diet as the primary environmental modulator of gut microbiota composition [39]. However, this hierarchical view oversimplifies the complex diet–exercise interactions observed in our data. Under NC conditions, both voluntary and forced exercise modestly increased alpha diversity, with forced exercise exerting a greater effect. Notably, the V-NC group exhibited the lowest diversity across all conditions, seemingly paradoxical given that exercise typically promotes microbial health [24,26].

This counterintuitive finding likely reflects the inherent limitations of voluntary exercise as an experimental model. Unlike forced treadmill training, which provides standardized intensity, duration, and frequency, voluntary wheel running varies considerably between individual animals in terms of total distance, bout duration, and circadian patterns. This behavioral heterogeneity translates to inconsistent physiological stimuli, including variable lactate production, differential sympathetic activation, and heterogeneous alterations in gut transit time, resulting in high inter-individual variability in microbial responses. Indeed, Allen et al. (2015) demonstrated that forced and voluntary exercise differentially alter gut microbiota composition and function, with forced exercise inducing more pronounced and consistent changes in bacterial community structure [26].

These findings underscore that diet and exercise do not act hierarchically but rather interact synergistically to shape gut microbial ecology. While HFD predominantly drove overall community structure (as evidenced by beta-diversity clustering), exercise modality critically determined the abundance of specific functional guilds and particularly beneficial genera such as *Akkermansia*, which emerged exclusively in exercised HFD groups. Thus, exercise effects are highly context-dependent, with both diet composition and exercise modality determining microbial outcomes.

At the phylum level, four taxa constituted the core microbiome (Figure 3): Firmicutes, Bacteroidota, Actinobacteria, and Deferribacterota. Firmicutes dominated across all groups, reflecting their fundamental role in mammalian gut ecosystems [40,41]. HFD significantly reduced the abundance of Firmicutes, likely through competitive exclusion by lipid-specialized phyla, while exercise exerted minimal effects on Firmicutes levels, consistent with previous rodent studies [26,27,42].

Bacteroidota, essential for protein and lipid catabolism, decreased significantly in V-NC compared to C-NC but remained stable across HFD groups, contrasting with reports of HFD-induced Bacteroidota reductions [43]. The Firmicutes:Bacteroidota (F:B) ratio has been proposed as a metabolic health biomarker, with elevated ratios associated with obesity. However, Magne et al. (2020) conclusively demonstrated that this ratio lacks diagnostic utility due to inconsistent associations across populations and by confounding other compositional factors [44]. Our data support this conclusion, as the F:B ratio showed poor correspondence with metabolic and weight outcomes.

Proteobacteria, a phylum enriched in dysbiotic states, increased markedly in HFD groups with minimal NC presence, consistent with HFD-induced expansion of Gram-negative facultative anaerobes [43]. Most notably, *Verrucomicrobia*, represented exclusively by *Akkermansia muciniphila*, emerged only in exercised HFD groups. This exercise-dependent enrichment aligns with studies demonstrating physical activity-associated *Akkermansia* expansion [45,46] and reflects this bacterium’s capacity to utilize exercise-induced metabolites while conferring metabolic benefits, including improved glucose homeostasis and intestinal barrier integrity [47]. *Deferribacterota* abundance also increased under HFD, consistent with its association with high-fat feeding [48].

At the genus and species levels, distinct diet–exercise interaction patterns emerged. NC groups were characterized by elevated *Lactobacillus*, *Kineothrix*, and *Eisenbergiella*, genera commonly associated with fiber-rich diets and carbohydrate fermentation [49,50,51]. Specifically, *Kineothrix alysoides* and *Wautersiella intestinalis* predominated in NC-fed animals, reflecting their roles in fiber degradation and intestinal barrier maintenance, respectively [14]. In contrast, HFD groups were dominated by *Peptococcus* (*P. niger* and *P. simiae*) and *Romboutsia ilealis*, consistent with lipid-enriched microbial communities [52,53]. Notably, forced exercise under HFD conditions (F-HFD) promoted *Ruthenibacterium lactatiformans* and *Desulfonispora thiosulfatigenes*, fermentative species associated with metabolite production that are beneficial for gut health, whereas voluntary exercise produced less consistent taxonomic shifts, likely reflecting the variability in exercise intensity between individual animals [26].

Beyond taxonomic composition, the interaction between diet and exercise significantly shaped the functionally relevant microbial genera, particularly those involved in host metabolism. Short-chain fatty acids (SCFAs), including acetate, propionate, and butyrate, are microbial metabolites produced through the fermentation of dietary substrates and play central roles in metabolic regulation, immune modulation, and the maintenance of gut barrier integrity [13,54]. In the present study, several established SCFA-producing genera, *Coprococcus*, *Butyricicoccus*, *Blautia*, *Anaerostipes*, *Oscillospiraceae incertae sedis*, and *Roseburia*, were significantly modulated by both diet and exercise. While HFD alone increased the abundance of select SCFA producers, structured exercise—particularly forced treadmill exercise under HFD conditions—elicited the most pronounced enrichment, with the F-HFD group exhibiting the highest relative abundance of these taxa.

Functional guild analysis revealed how exercise-diet synergies shape metabolically relevant taxa. F-HFD significantly enriched SCFA-producing genera, including *Coprococcus*, *Blautia*, and *Roseburia*, which are all bacteria that enhance gut barrier integrity, immune regulation, and metabolic function through butyrate and propionate production [54,55]. *Blautia*, typically associated with high-fiber diets [56], increased under both HFD and exercise conditions, with forced exercise exerting the greatest effect, confirming previous observations [33,57]. Gut barrier-associated genera exhibited divergent responses. *Lactobacillus*, typically fiber-responsive, decreased markedly under both HFD and exercise conditions, which is consistent with multiple studies reporting exercise-associated *Lactobacillus* reductions [36,37,57,58], while *Akkermansia* and *Dorea* increased exclusively in exercised HFD groups. Most critically, forced exercise mitigated HFD-induced pathogenic enrichment, and potentially pathogenic bacteria (*Shigella*, *Escherichia*, *Proteus*) were significantly less abundant in F-HFD compared to C-HFD and V-HFD groups. This suggests that structured exercise promotes a protective microbial profile capable of partially compensating for diet-induced dysbiosis [26,55].

*Akkermansia muciniphila* emerged as a key biomarker of exercise–diet interactions. This mucin-degrading bacterium of the Verrucomicrobia phylum appeared exclusively in exercised HFD groups (V-HFD: 2.56% and F-HFD: 1.65%), demonstrating exercise-dependent enrichment under metabolically challenging dietary conditions. *Akkermansia* confers multiple metabolic benefits, including improved glucose homeostasis, enhanced intestinal barrier integrity, and anti-inflammatory effects through mucin-derived metabolite production [59,60]. Our findings align with numerous studies documenting exercise-induced *Akkermansia* expansion in both animal models and human athletes [24,33,46,57,58,61]. Critically, we observed dose-dependent relationships (Figure 5): forced exercise distance correlated positively with *Akkermansia* abundance (r = 0.68, *p* < 0.001), while baseline body weight correlated negatively (r = −0.51, *p* < 0.01), which suggests that *Akkermansia* could be a potential associative mediator between structured exercise, metabolic status, and weight regulation. These findings suggest exercise intensity, rather than voluntary activity alone, as the primary driver of *Akkermansia* enrichment. From a translational perspective, these findings suggest that structured, moderate-intensity exercise may be more effective than self-directed physical activity in mitigating diet-induced gut dysbiosis. This may have implications for exercise prescription strategies in individuals consuming obesogenic diets, where structured programs with defined intensity, frequency, and duration—consistent with the FITT principle [22]—could offer greater microbiome-related benefits than general recommendations to increase physical activity. However, direct translational studies in human populations are needed to confirm these observations. While forced exercise produced the most pronounced beneficial microbial shifts, it should be recognized that this modality may also induce stress-related physiological adaptations with both beneficial and adverse consequences for the host.

Overall, the present findings indicate that structured moderate-intensity exercise may offer a more consistent strategy than voluntary activity for mitigating high-fat diet–associated gut microbiota dysbiosis. Nevertheless, although forced exercise induced the most pronounced microbial changes, this modality may also evoke stress-related physiological responses that could have both beneficial and adverse implications for the host. Therefore, the translational relevance of forced treadmill paradigms must be interpreted cautiously, particularly given that neuroendocrine activation may independently influence microbial remodeling. Direct translational studies in human populations are needed to confirm these observations. From a practical perspective, the results highlight the importance of structured exercise programs for individuals exposed to obesogenic diets, where training parameters aligned with the FITT principle may help optimize metabolic and gut microbiota responses [22], could offer greater microbiome-related benefits than general recommendations to increase physical activity. Additional research in human populations will be required to determine whether these microbiome responses to exercise are reproducible in clinical settings. Notably, our findings were consistent across both male and female rats, suggesting that the effects of diet and exercise modality on gut microbiota composition are not sex-dependent in this model, which strengthens the generalizability of the observed results.

Future studies incorporating longer intervention periods, serial fecal sampling to capture temporal microbiome dynamics, direct functional measurements (e.g., SCFA quantification, inflammatory cytokines, and gut permeability markers), and integrated metabolomic and metatranscriptomic analyses would further elucidate the mechanistic pathways underlying exercise-mediated microbial remodeling [58,62]. Additionally, translational studies in human populations are needed to determine whether structured exercise programs, designed according to the FIIT principle, can produce comparable microbiome benefits in individuals consuming obesogenic diets [22,40]. Characterization of mucosal-associated intestinal microbiota, in addition to luminal communities, would provide further insight into host-microbe interactions at the tissue level and represent a valuable avenue for future investigation.

This study employed full-length 16S rRNA gene sequencing with strict classification criteria (≥97% identity and ≥95% coverage), providing a robust taxonomic resolution that surpasses conventional short-read amplicon approaches. While this enabled confident genus- and species-level identification, integration of metagenomic or metatranscriptomic approaches in future studies would further enable direct functional characterization of the observed microbial shifts. Similarly, although our taxonomic findings strongly suggest enrichment of SCFA-producing and gut barrier-associated genera in exercised groups, direct quantification of functional readouts such as SCFA concentrations, gut permeability markers, and inflammatory cytokines would provide complementary mechanistic evidence, representing a priority for future investigation. Sperman’s correlation analyses were conducted to explore associations between *Akkermansia* and the exercise method; however, these analyses were exploratory in nature, and formal false discovery rate (FDR) correction was not applied, which may increase the risk of type one error. Regarding exercise modality, forced treadmill exercise delivered the most consistent and pronounced microbial benefits; however, this modality may also elicit a neuroendocrine stress response (e.g., elevated corticosterone), and future studies incorporating stress biomarker measurements would help disentangle exercise-specific effects from stress-related contributions to microbial remodeling. Furthermore, body weight was only recorded at the beginning and the end of the intervention, and additional metabolic parameters such as food intake, glucose tolerance, and adiposity indices were not measured. Future studies incorporating longer intervention periods, serial fecal sampling to capture temporal microbiome dynamics, direct functional measurements (e.g., SCFA quantification, inflammatory cytokines, gut permeability markers), and integrated metabolomic and metatranscriptomic analyses would further elucidate the mechanistic pathways underlying exercise-mediated microbial remodeling [58,62]. Additionally, translational studies in human populations are needed to determine whether structured exercise programs, designed according to the FITT principle, can produce comparable microbiome benefits in individuals consuming obesogenic diets [22,24].

## 5. Conclusions

This study demonstrates that dietary composition and exercise modality independently and synergistically shape gut microbial communities in rats. Although HFD increased overall microbial diversity, it promoted a dysbiotic profile characterized by the enrichment of potentially pathogenic taxa. In contrast, structured forced exercise markedly attenuated HFD–induced dysbiosis, promoting the expansion of metabolically beneficial genera, including *Akkermansia*, *Blautia*, *Coprococcus*, and *Roseburia*, which are associated with improved metabolic regulation and gut barrier integrity. Notably, *Akkermansia muciniphila* emerged exclusively in exercised high-fat diet groups and exhibited a positive association with exercise intensity and a negative association with body weight, supporting its potential role as a biomarker of exercise-mediated metabolic benefit.

Collectively, these findings underscore exercise modality as a critical determinant of gut microbiome responses to diet, extending beyond the effects of physical activity alone. While diet remains the primary driver of overall microbial structure, exercise—particularly when standardized and sufficiently intense—can selectively modulate key functional taxa and partially offset diet-induced dysbiosis. While these findings are strengthened by the use of both male and female rats, full-length 16S rRNA sequencing with strict classification criteria, well-controlled exercise paradigms, future studies incorporating direct functional measurements such as SCFA quantification and inflammatory markers, and integrated metabolomic and metatranscriptomic analyses would further elucidate the mechanistic pathways underlying the observed exercise-mediated microbial shifts. From a clinical standpoint, these results support the recommendation that structured exercise programs with controlled intensity and frequency should be considered as a complementary strategy alongside dietary interventions for the management of gut dysbiosis and associated metabolic disorders. Such insights may ultimately inform precise nutrition and exercise strategies for the prevention and management of metabolic disorders.

## Figures and Tables

**Table 1:** Experimental design and rat allocation per study group.

Diet Type	Exercise Status	Total
NC	C	12
	V	9
	F	10
HFD	C	10
	V	12
	F	10
Total		63

**Table 2:** Alpha diversity as measured by Shannon index and Sobs value.

Group	Shannon Index	Sobs Analysis
C-NC	2.94 ± 0.15	431.2 ± 103.1
V-NC	2.76 ± 0.33	299.7 ± 149.8
F-NC	2.88 ± 0.22	459.7 ± 181.4
C-HFD	3.47 ± 0.34	529.7 ± 167.4
V-HFD	3.63 ± 0.19	451.9 ± 116.8
F-HFD	3.61 ± 0.18	547.7 ± 170.2

C-NC: Control–normal chow; V-NC: Voluntary exercise–normal chow; F-NC: Forced exercise–normal chow; C-HFD: Control–high-Fat diet; V-HFD: Voluntary exercise–high-fat diet; and F-HFD: Forced exercise–high-fat diet. Values are represented as the mean ± standard deviation.

**Table 3:** The gut microbial mean relative abundance at the phylum level of selected phyla.

Phylum	C-NC	V-NC	F-NC	C-HFD	V-HFD	F-HFD
Bacteroidota	1.47	0.33 (0.03 *)	1.13 (1.00 *)	0.96	2.05 (0.79 *)	1.42 (1.00 *)
Firmicutes	96.83	98.04 (0.74 *)	97.96 (0.91 *)	88.77	86.52 (0.98 *)	90.71 (0.99 *)
Proteobacteria	0.03	0.06	0.03	4.15 (**2.4 × 10^−7 †^**)	6.31 (**0.0007 ^†^**)	1.94 (**1.3 × 10^−6 †^**)
Verrucomicrobia	0.00	0.00 (1.00 *)	0.00 (1.00 *)	0.00	2.56 (7.47 × 10^−5^ *)	1.65 (1.56 × 10^−11^ *)

* Compares the diet groups (V-NC and F-NC to C-NC and V-HFD and F-HFD to C-HFD). ^†^ Compares the exercise groups (C-HFD to C-NC, V-HFD to V-NC, and F-HFD to F-NC). The underline shows the phylum with the highest relative abundance. The *p*-values are shown in the brackets, and the bold font shows significance (*p* < 0.05).

**Table 4:** Relative abundance (%) of gut bacterial genera involved in SCFA production, gut barrier integrity, and potential pathogenicity across diet and exercise groups with corresponding statistical significance (ANOVA ART).

Genus	Group	C-NC	V-NC	F-NC	C-HFD	V-HFD	F-HFD	*p*-Value
*Coprococcus*	SCFA	0.01	0.03	0.07	0.1	0.78	3.03	1 × 10^−9^
*Blautia*	SCFA	1.47	1.37	1.85	2.03	6.34	7.65	7 × 10^−3^
*Roseburia*	SCFA	0.14	0.18	0.19	1.38	0.84	0.5	2 × 10^−2^
*Dorea*	Gut barrier	0.01	0	0.01	0.45	0.47	1.3	1 × 10^−3^
*Akkermansia*	Gut barrier	0	0	0	0	2.56	1.65	3 × 10^−2^
*Lactobacillus*	Gut barrier	18.06	15.62	4.12	4.31	0.09	0.01	3 × 10^−2^
*Proteus*	Potentially Pathogenic	0	0	0	0.48	0.02	0	3 × 10^−5^
*Escherichia*	Potentially Pathogenic	0.01	0.02	0	2.18	3.4	0	4 × 10^−3^
*Shigella*	Potentially Pathogenic	0.01	0.01	0	1.27	2.04	0	1 × 10^−2^

**Table 5:** Relative abundance (%) of potentially pathogenic species across diet and exercise groups.

Species	C-NC	V-NC	F-NC	C-HFD	V-HFD	F-HFD	*p*-Adj
*Shigella flexneri*	0.00	0.00	0.00	0.89	1.45	0.00	0.004
*Escherichia fergusonii*	0.00	0.00	0.00	2.09	3.23	0.00	0.004
*Shigella sonnei*	0.00	0.00	0.00	0.38	0.58	0.00	0.006
*Proteus mirabilis*	0.00	0.00	0.00	0.47	0.00	0.00	1.45 × 10^−5^
*Escherichia coli*	0.00	0.00	0.00	0.08	0.15	0.00	0.003

C-NC: Control–normal chow; V-NC: Voluntary exercise–normal chow; F-NC: Forced exercise–normal chow; C-HFD: Control–high-fat diet; V-HFD: Voluntary exercise–high-fat diet; F-HFD: Forced exercise–high-fat diet. *p*-adj: adjusted *p*-value from ART ANOVA.

## Data Availability

The sequencing data presented in this study are openly available in the NCBI Sequence Read Archive under BioProject accession number PRJNA1231772 (https://www.ncbi.nlm.nih.gov/bioproject/PRJNA1231772, accessed on 1 December 2025). Additional processed data supporting the findings of this study are available upon reasonable request.

## Supplementary Materials

The following supporting information can be downloaded at https://www.mdpi.com/article/10.3390/nu18050847/s1, Table S1: Top five most abundant bacterial genera per experimental group, ranked by mean relative abundance (%); Table S2: Top five most abundant bacterial species per experimental group, ranked by mean relative abundance (%).
