# Supplementary material for: Structured Exercise Modulates Gut Microbiota Composition and Protects Against Diet-Induced Dysbiosis in a Rat Model
Source: Nutrients. 2026 Mar 5;18(5):847. doi: 10.3390/nu18050847 (PMC12987009; doi:10.3390/nu18050847)
Supplement: Supplementary file 1 [file nutrients-18-00847-s001.zip › nutrients-4147100-supplementary.pdf]

Supplementary Table S1. Top five most abundant bacterial genera per experimental group, ranked by mean relative abundance (%).

Genus	Group	Mean relative abundance
Lactobacillus	C_NC	18.06
Kineothrix	C_NC	17.44
Eisenbergiella	C_NC	14.95
Waltera	C_NC	14.52
Lacrimispora	C_NC	12.47
Kineothrix	V_NC	18.33
Eisenbergiella	V_NC	16.47
Lactobacillus	V_NC	15.62
Waltera	V_NC	14.41
Lacrimispora	V_NC	11.33
Kineothrix	F_NC	22.58
Waltera	F_NC	20.61
Eisenbergiella	F_NC	19.10
Lacrimispora	F_NC	14.31
Lactobacillus	F_NC	4.12
Peptococcus	C_HFD	19.58
Romboutsia	C_HFD	11.55
Kineothrix	C_HFD	5.89
Mucispirillum	C_HFD	5.09
Lactobacillus	C_HFD	4.31
Peptococcus	V_HFD	13.60
Kineothrix	V_HFD	6.39
Blautia	V_HFD	6.34
Romboutsia	V_HFD	4.01
Eisenbergiella	V_HFD	3.78
Peptococcus	F_HFD	17.41
Romboutsia	F_HFD	10.35
Blautia	F_HFD	7.65
Eisenbergiella	F_HFD	5.55
Ruthenibacterium	F_HFD	4.90

Supplementary Table S2. Top five most abundant bacterial species per experimental group, ranked by mean relative abundance (%).

Species	Group	Mean relative abundance
Kineothrix alysoides	C_NC	17.45
Waltera intestinalis	C_NC	14.52
Lactobacillus johnsonii	C_NC	10.14
Lacrimispora saccharolytica	C_NC	9.86
Eisenbergiella tayi	C_NC	7.38
Kineothrix alysoides	V_NC	18.33
Waltera intestinalis	V_NC	14.41
Lacrimispora saccharolytica	V_NC	9.10
Eisenbergiella tayi	V_NC	8.45
Lactobacillus johnsonii	V_NC	8.44
Kineothrix alysoides	F_NC	22.58
Waltera intestinalis	F_NC	20.61
Lacrimispora saccharolytica	F_NC	11.29
Eisenbergiella tayi	F_NC	10.20
Eisenbergiella massiliensis	F_NC	6.50
Peptococcus niger	C_HFD	13.32
Romboutsia ilealis	C_HFD	10.51
Peptococcus simiae	C_HFD	6.28
Kineothrix alysoides	C_HFD	5.90
Mucispirillum schaedleri	C_HFD	5.09
Peptococcus niger	V_HFD	9.14
Kineothrix alysoides	V_HFD	6.40
Peptococcus simiae	V_HFD	4.47
Romboutsia ilealis	V_HFD	3.66
Ligilactobacillus murinus	V_HFD	3.58
Peptococcus niger	F_HFD	11.73
Romboutsia ilealis	F_HFD	9.27
Peptococcus simiae	F_HFD	5.69
Ruthenibacterium lactatiformans	F_HFD	4.91
Desulfonispora thiosulfatigenes	F_HFD	3.56
